# Dynasore Protects Mitochondria and Improves Cardiac Lusitropy in Langendorff Perfused Mouse Heart

**DOI:** 10.1371/journal.pone.0060967

**Published:** 2013-04-15

**Authors:** Danchen Gao, Li Zhang, Ranvir Dhillon, Ting-Ting Hong, Robin M. Shaw, Jianhua Zhu

**Affiliations:** 1 Department of Cardiology, The First Affiliated Hospital, College of Medicine, Zhejiang University, Hangzhou, Zhejiang, China; 2 Cardiovascular Research Institute, University of California San Francisco, San Francisco, California, United States of America; 3 Department of Medicine, Division of Cardiology, University of California San Francisco, San Francisco, California, United States of America; UAE University, United Arab Emirates

## Abstract

**Background:**

Heart failure due to diastolic dysfunction exacts a major economic, morbidity and mortality burden in the United States. Therapeutic agents to improve diastolic dysfunction are limited. It was recently found that Dynamin related protein 1 (Drp1) mediates mitochondrial fission during ischemia/reperfusion (I/R) injury, whereas inhibition of Drp1 decreases myocardial infarct size. We hypothesized that Dynasore, a small noncompetitive dynamin GTPase inhibitor, could have beneficial effects on cardiac physiology during I/R injury.

**Methods and Results:**

In Langendorff perfused mouse hearts subjected to I/R (30 minutes of global ischemia followed by 1 hour of reperfusion), pretreatment with 1 µM Dynasore prevented I/R induced elevation of left ventricular end diastolic pressure (LVEDP), indicating a significant and specific lusitropic effect. Dynasore also decreased cardiac troponin I efflux during reperfusion and reduced infarct size. In cultured adult mouse cardiomyocytes subjected to oxidative stress, Dynasore increased cardiomyocyte survival and viability identified by trypan blue exclusion assay and reduced cellular Adenosine triphosphate(ATP) depletion. Moreover, in cultured cells, Dynasore pretreatment protected mitochondrial fragmentation induced by oxidative stress.

**Conclusion:**

Dynasore protects cardiac lusitropy and limits cell damage through a mechanism that maintains mitochondrial morphology and intracellular ATP in stressed cells. Mitochondrial protection through an agent such as Dynasore can have clinical benefit by positively influencing the energetics of diastolic dysfunction.

## Introduction

Heart failure is a major cause of morbidity and mortality in the United States [1], of which diastolic heart failure (DHF) is an important entity with rising prevalence. Of the 6 million patients with heart failure, as many as half had diastolic dysfunction [2], [3]. The one year mortality associated with hospitalization due to diastolic dysfunction is between 22 and 29% [4]. Myocardial ischemia is a major contributor to DHF. Acute ischemia can result in DHF due to rapid myocardial changes including edema, calcium accumulation, and inflammation [5], [6] and the severity of diastolic dysfunction depends on the duration of ischemia [7]. Hearts subjected to chronic microvascular or untreatable chronic ischemia also have diastolic dysfunction [4].

Current therapeutic approaches to DHF due to ischemia focus on relieving the ischemia with reperfusion [8]. Ironically, ischemia/reperfusion (I/R) can result in direct myocardial injury [9] and negatively affect diastolic function. In recent decades, the mechanisms involved in I/R injury have started to be identified. Cellular death and damage pathways involve subcellular organelles such as mitochondria which are critical mediators due to their ability to generate Adenosine triphosphate (ATP) and reactive oxygen species (ROS). During ischemia, progressive ATP depletion inhibits ion pump function which leads to intracellular accumulation of calcium [10], [11]. Also, reintroduction of oxygen during reperfusion will regenerate ATP, however, it will also damage the electron transport chain resulting in increased mitochondrial generation of ROS [12], [13]. Mitochondrial Ca^2+^ overload [14] and increased ROS result in opening of the mitochondrial permeability transition pore (MPT) [15], which initiates apoptosis and cell death by causing mitochondrial swelling and rupture. Interestingly, inhibition of MPT is reported to reduce infarct size [16].

More recently, the integrity and morphology of mitochondrial network has been recognized as critical to cell fate. In a healthy non-stressed intact cell, mitochondria consist of a continuous mitochondrial reticulum, which undergoes constant fusion and fission, two opposing processes controlled by local GTP gradients and mitochondrial energetics [17]. Dynamin-related GTPases such as mitofusins (MFN1, MFN2) and the mitochondrial inner membrane optic atrophy protein 1 (OPA1) isoforms are pro-fusion. Alternatively, scission requires the pro-fission multimers containing mitochondrial fission protein 1 (FIS1), Mitochondrial fission factor (MFF), and dynamin related protein 1 (Drp1). The fine balance between mitochondria fusion and fission determines cell fate [17]. Cardiac I/R injury results in significant mitochondrial fission, which induces apoptotic cell death. Inhibition of mitochondrial fission mediated by Drp1 can limit infarct size in I/R injury [18].

A critical yet unaddressed issue is whether mitochondrial protection limits ischemia related diastolic dysfunction. Dynasore is a cell-permeable small molecule that non-competitively inhibits the GTPase activity of dynamin1, dynamin2 and Drp1 [19]. We found that low dose Dynasore significantly preserves lusitropy of ex vivo perfused hearts subject to I/R injury. Dynasore also increases cardiomyocyte survival, and decreases cellular ATP consumption in stressed cardiomyocytes. In support of mitochondrial protection, we found that low dose Dynasore is sufficient to prevent oxidative stress induced mitochondrial fission in cultured cells. Thus small molecule based Drp1 inhibition is a potential therapeutic approach to ischemia related DHF.

## Materials and Methods

### Guidelines for Animal Research

This study was approved by the University of California San Francisco Committee on the Use and Care of Animals (IACUC). All procedures used in this study are in agreement with the guidelines of the University of California San Francisco IACUC. Animals were housed in UCSF facility of Laboratory Animal Resource Center. All investigation conformed to the Guide for the Care and Use of Laboratory Animals published by the US National Institutes of Health (NIH Publication no. 85-23, received 1996).

### Materials

**Figure 1:**
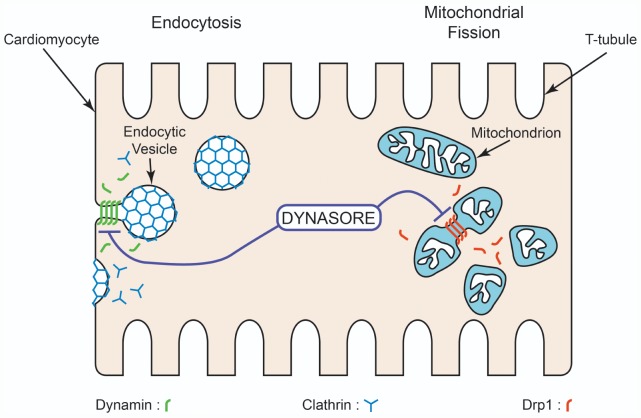
Cellular Targets of Dynasore. Dynasore is a specific small molecular GTPase inhibitor that targets Dynamin1 and Dynamin2 which are responsible for pinching off endocytic vesicles, and Drp1 which is responsible for mitochondrial fission.

Unless otherwise stated, Dynasore (Figure 1) and all other materials were purchased from Sigma Chemical (St. Louis, MO).

### Langendorff-Perfused Heart

Male C6/Black mice (8∼12 weeks; Charles River) were anesthetized with isoflurane (flow 3%) and 100% O_2_ in an anesthesia chamber and anti-coagulated with heparin (50 IU, i.p.). After cervical dislocation, hearts were rapidly excised, mounted on a Langendorff apparatus (ADInstrument, Colorado Springs, CO) and perfused retrogradely [20], [21] at a constant rate of 2.6 ml/min with oxygenated Krebs-Henseleit buffer containing (mmol/L): NaCl 118, NaHCO_3_ 24, CaCL_2_.2H_2_O 2.5, KCL 4.7, KH_2_PO_4_ 1.2, MgSO_4_-7H_2_O 1.2, Glucose 11, EDTA 0.5, adjusted to a pH of 7.4 [22], [23]. The apparatus was water-jacketed for temperature control to maintain a core temperature of the heart at 37°C. The buffer passed through a membranous “lung” made of SilasticTM Medical Grade Tubing, which was gassed continuously with 95% O_2_-5% CO_2_. Fine platinum electrodes were placed on the right atrium and apex of the left ventricle to record the electrocardiogram and heart rate throughout the experiment. A Millar MIKRO-TIP catheter transducer (Millar Instruments, Houston, TX) was inserted into the left ventricle from the left atrium to measure left ventricular pressure. Left ventricular end diastolic pressure (LVEDP), left ventricular end systolic pressure (LVESP) and heart rate were monitored and recorded continuously using PowerLab system (ADInstruments). Left ventricular developed pressure (LVDP) was calculated by subtracting LVEDP from LVESP. Hearts were paced at 360 bpm with bipolar electrodes attached to the right atrium, using stimuli delivered from a stimulator (ADInstruments Colorado Springs, CO).

After the initial 15 min stabilization, hearts were excluded from further study if they exhibited one or more of the following exclusion criteria: LVEDP higher than 20 mmHg; LVDP less than 50 mmHg; intrinsic heart rate less than 280 bpm or irregular; or aortic regurgitation. The volume of the perfusate was reduced to 200 ml and allowed to recirculate. The hearts were then randomized to one of the following two treatment groups: Dynasore group (n = 8, added into the recirculating perfusate in stepwise fashion to reach a final concentration of 1 µM within 120 min of recirculation) or DMSO control group (n = 8, added in a similar manner of Dynasore). Hearts were then subjected to 30 min of global ischemia followed by 1 hour of reperfusion. Pacing was initiated after stabilization except during ischemia and was reinitiated 2 min after reperfusion.

### Myocardial cTnI Release

Cardiac effluent was collected from apex at baseline, before ischemia and during reperfusion. The samples were snap frozen immediately in liquid nitrogen and stored at −80°C for later analysis. Cardiac effluent samples from 5 hearts in each group were then used to determine cardiac specific troponin I (cTnI) concentration using a commercial cTnI ELISA kit (Life Diagnostics, Inc., West Chester, PA).

### Determination of Infarct Size

Propidium iodide (excitation, 535 nm; emission, 617 nm) was used to determine infarct size according to a previously established method [24], [25] with modest modification. Briefly, at final 15 min reperfusion period, 300 µg of propidium iodide (Sigma Chemical, St. Louis, MO) was injected into the right atrium and allowed to perfuse through the coronary vasculature. At the end of reperfusion, hearts were then removed from the apparatus, sliced perpendicularly to the long axis of the heart into 4∼5 equal-thickness transverse slices. The top and bottom surfaces of each slice were imaged by widefield epifluorescence microscopy with a Cy3 filter at an exposure of 500 ms per frame, and grayscale images were captured using a sensitive CCD camera with white pixels corresponding to PI positive signals. Total left ventricular area and infarct area for each image were analyzed using ImageJ software. Infarct size was calculated by total infarct area summed from all the slices and expressed as percentage of the total left ventricular area.

### Isolation and Culture of Adult Mouse Cardiomyocytes

Mouse ventricular myocytes were isolated from male adult C6/Black mouse (8∼12 weeks; Charles River) after dissociation with collagenase II (2 mg/ml, Worthington, Lakewood, NJ) using a previously described method [26], [27]. After dissociation, cardiomyocytes were plated on laminin-precoated 35 mm^2^ culture dishes at a density of ∼1,500/mm^2^ and maintained in a humidified atmosphere of 5% CO_2_ at 37°C. After 1 hour of plating, cardiomyocytes were replenished with fresh medium (serum supplemented or depleted) and subjected to 2 hours of drug treatment (Dynasore or vehicle) followed by oxidative stress (30 µM H_2_O_2_ for 35 min). For ATP supplement experiments, the cells were treated with 3 mM ATP for 30 min before exposure to H_2_O_2_.

### In vitro Cardiomyocyte Survival and Viability Assay

After exposure to 30 µM H_2_O_2_ for 35 min [27], cardiomyocyte survival and viability were analyzed by trypan blue exclusion (TBE) assay, which is a method to determine cell survival and changes in cell morphology in experimental models [28], [29]. In brief, cardiomyocytes were stained with 0.04% (w/v) trypan blue solution (Gibco, Invitrogen, Carlsbad, CA) at room temperature for 7 min. When cell membranes are irreversibly damaged, the anionic dye trypan blue is taken up by dead cells. Cardiomyocytes were then visualized at 40× magnification by microscopy. For each experiment, a total of 200 cardiomyocytes were analyzed from 10 different fields/dish. Cells that excluded trypan blue (TBEs) were considered to have survived. Healthy rod-shaped myocytes (rods) were identified when the length/width ratio was >3∶1 [30]. Contracted cells were defined when the length/width ratio was <3∶1. Trypan blue-positive cells were identified when the trypan blue was present irrespective of whether the cells were rod-shaped or contracted [28], [31]. Morphologic changes (viability) were measured by determining the number of rods relative to all TBEs (rods and contracted cells) of the 200 cardiomyocytes analyzed. The percent survival and viability were calculated as follow:
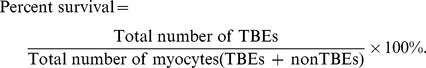



Note total number of myocytes (TBEs+non-TBEs) = 200.
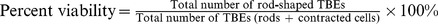



### ATP Measurement

A luminescence assay (Promega, Madison, WI) was used to quantify cardiomyocyte and Hela cell ATP content [32]. Briefly, after Dynasore treatment and H_2_O_2_ exposure, cardiomyocytes were lysed and ATP content was measured in the cell lysates. Meanwhile, in a separate set of wells following same experimental protocol, surviving cardiomyocytes were counted using a TBE assay. Cellular ATP per single live cardiomyocyte was then calculated for each treatment condition. Similar procedures were applied to cultured non-stressed Hela cells treated with control or Dynasore.

### Live-cell Mitochondria Imaging with Spinning Disc Confocal Microscopy

HeLa cells (ATCC, Manassas, VA) were maintained in DMEM (Invitrogen) supplemented with 10% FBS (Invitrogen) and 100 µg/ml Normocin (Amaxa, Lonza Walkersville Inc, Walkersville, MD). Cells were maintained in a humidified atmosphere of 5% CO_2_ at 37°C. Cells were seeded at a density of 7 × 10^4^ cells/cm^2^ and allowed to adhere overnight. Cells were then transduced with Organelle Lights™ Mito-RFP *BacMam 1.0 (Invitrogen). Twenty-four hours after transduction, cells were pretreated with either control or 1 µM Dynasore for 1 hour before being exposed to normal conditions or 200 µM H_2_O_2_ for 15 minutes. Before and after exposure to H_2_O_2_, cells were imaged using a Nikon T*i* inverted microscope, Yokogowa CSU-X1 spinning disk confocal unit with 568-nm DPSS laser source, and a high resolution Cool SNAP HQ^2^ camera (Photometrics, Tucson, AZ). Images were acquired at 400 ms exposure per frame and automatically processed using a *bas relief* filter to highlight edges.

### Statistical Analysis

Statistical analysis was performed using GraphPad Prism 5. The data are expressed as means ± SEM. Difference between control and experimental groups were determined using a one-way analysis of variance (ANOVA) for multiple groups. Difference between every two groups was determined using Bonferroni post hoc test. For comparison between two groups with timed repeated measurements, a two-way ANOVA (treatment and time are considered as the two variances) was used. P<0.05 was considered to be significant.

## Results

### In Langendorff Perfused Mouse Hearts, Dynasore Prevents Pathologic LVEDP Elevation Following I/R Injury

**Figure 2:**
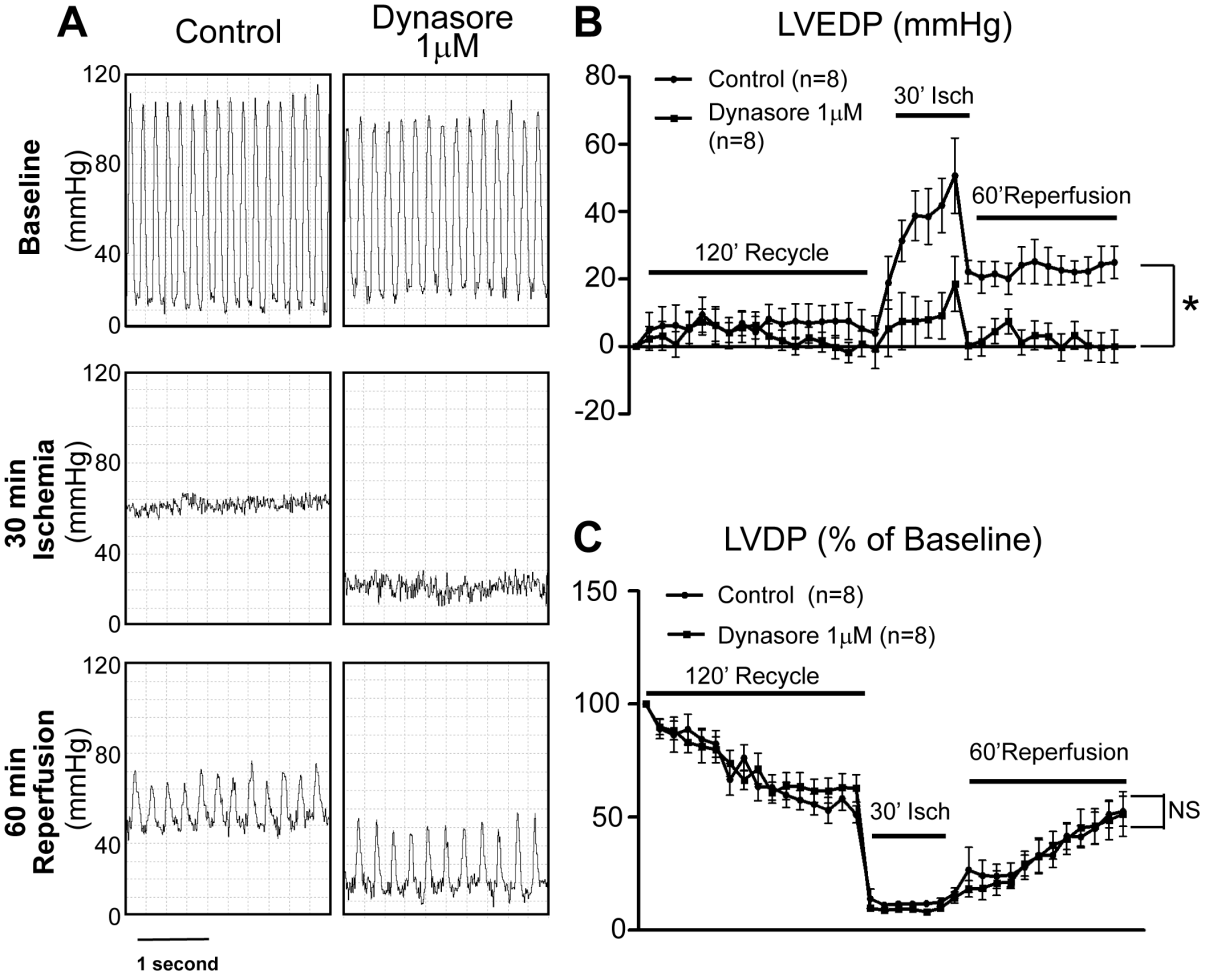
The cardiac lusitropic effect of Dynasore. Effect of 1 µM Dynasore pretreatment on ventricular function was studied in Langendorff perfused mouse hearts subjected to 30 min no-flow global ischemia followed by 60 min reperfusion. Hearts were paced at 360 bpm during the whole experimental protocol except the ischemia period and pacing was reinitiated at 2 min into the reperfusion period. A. Representative Left ventricular pressure tracing in a control heart and a Dynasore treated heart. B, C; Left ventricular end diastolic pressure (LVEDP, B) and left ventricular developed pressure (LVDP, C) are summarized and compared between control group and Dynasore group. (* *P*<0.05).

We investigated the effects of Dynasore on intracardiac pressure during I/R injury. Using Langendorff perfusion, hearts were subjected to no-flow global ischemia followed by restoration of flow, with and without the presence of Dynasore during the entire period. Results are in Figure 2A which contains ventricular pressure tracings at baseline, during ischemia, and post reperfusion from two representative hearts treated with vehicle or 1 µM of Dynasore, which was determined as a safe, complication free, cardiac protective dose based on a pilot study testing the pharmacological dose response curve of Dynasore (0.1–10 µM). As indicated, 30 min of global ischemia caused a significant elevation of LVEDP, which only had partial recovery after reperfusion. In addition, global ischemia resulted in a significantly drop of cardiac systolic function indicated by left ventricular developed pressure (LVDP), which returned to its pre-ischemia baseline after 60 min of reperfusion. The concurrent increase of LVEDP with a drop of LVDP indicates that a severe hypo-contractile state is initiated after ischemia and persists into reperfusion. However, pretreatment with 1 µM Dynasore prevented elevation of LVEDP without a change of LVDP. The summarized ventricular pressure data from eight independent experiments of each condition are presented in Figure 2B (LVEDP), Figure 2C (LVDP) and Table 1. Note a significant decrease of LVEDP in Dynasore treated hearts is present during the entire ischemia and reperfusion period. Dynasore’s beneficial effect on LVEDP and not LVDP indicates it benefits lusitropy more than it does on inotropy.

**Table 1:** Effect of 1 µM Dynasore pretreatment on ventricular function in Langendorff perfused mouse hearts during ischemia/reperfusion injury.

	LVEDP	(mmHg)	LVESP	(mmHg)	LVDP	(% of BL)
	Control	Dynasore	Control	Dynasore	Control	Dynasore
	(N = 8)	(N = 8)	(N = 8)	(N = 8)	(N = 8)	(N = 8)
**Baseline**	4.9±5.3	2.4±3.5	62.0±16.6	62.8±14.1	100.0±0.0	100.0±0.0
**Post-drug**	5.5±5.5	0.8±3.8	42.8±6.3	48.2±6.8	51.1±3.8	62.8±6.0
**Ischemia**						
**15 min**	31.4±6.1	7.5±8.5*	48.0±17.1	15.2±6.9	11.7±1.0	9.3±1.3
**30 min**	41.9±8.1	9.1±6.9***	57.8±15.4	24.2±9.8	12.4±1.8	9.8±1.0
**Reperfusion**						
**15 min**	21.6±3.7	4.6±3.7*	45.1±12.3	32.0±10.0	23.9±5.3	20.9±5.4
**30 min**	25.2±6.6	3.3±4.3	56.6±18.3	40.7±15.2	33.2±7.6	32.5±7.4
**60 min**	24.9±4.8	0.0±4.8*	64.7±20.0	46.1±6.2	52.6±6.7	51.4±9.9

* , ***indicate *P*<0.05, *P*<0.001 when compared between the two treatment groups.

LVEDP, left ventricular end diastolic pressure; LVESP, left ventricular end systolic pressure; BL, baseline.

### In I/R Injured Mouse Hearts, Dynasore Decreases Myocardial Death

**Figure 3:**
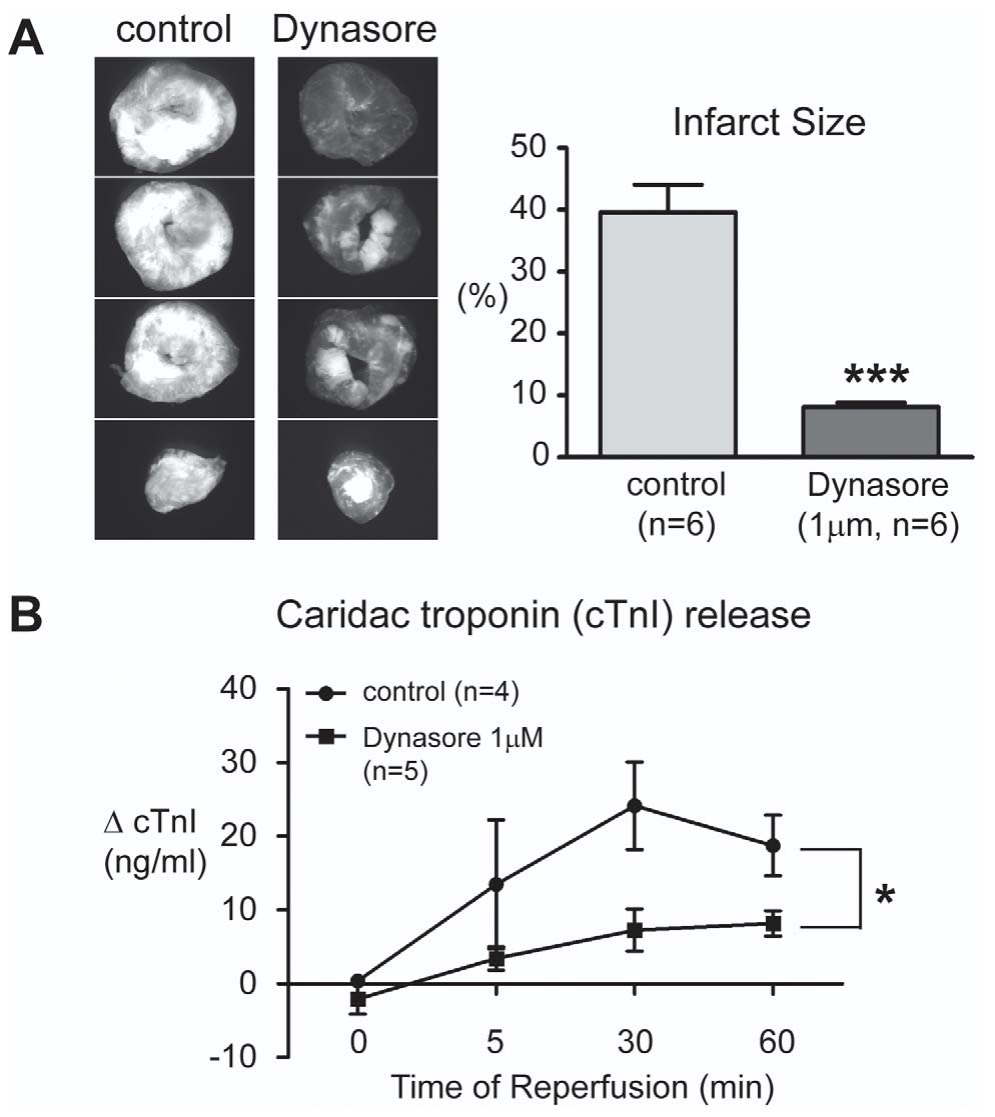
Dynasore decreases cardiomyocyte death in I/R injured mouse hearts. A. Myocardial infarct size was analyzed by Propidium Iodide (PI) perfusion. Left, representative fluorescence images of PI staining in a control and Dynasore treated hearts subjected to I/R injury. Right, average infarct size is presented as percentage over total left ventricular area and compared between the two treatment groups. B. Dynasore decreases cardiac troponin I (cTnI) efflux. Myocardial damage was evaluated by measurement of the release of cTnI in the coronary effluent during the 60 min reperfusion period. (* *P*<0.05, ***P<0.001).

Improved lusitropy implies less stress and ischemia related damage to the myocardium. We investigated myocardial damage with two separate direct assays: staining hearts for cardiomyocyte death and measuring troponin release. Propidium iodide (PI), a nuclear fluorescent dye, permeates through the damaged plasma membrane of cardiomyocytes that are at the early stage of cell death. As seen in the left panel of Figure 3A, the PI signal and hence cell death in heart slices from non-treated hearts is significantly higher. The PI positive area was traced in ImageJ and presented as percentage of total ventricular area. Infarct size calculated using this method indicates that, in control hearts, 30 min of ischemia followed by 1 hour of reperfusion results in 39.6% of PI positive infarct area. Perfusion with Dynasore significantly reduces infarct size to 8.1% (80% reduction, Figure 3A).

Myocardial death was further evaluated by measuring cTnI in the cardiac efflux. As seen in Figure 3B, myocardial damage induced by I/R injury causes cTnI release into cardiac effluent, an event within an hour post reperfusion (clinical cTnI requires as much as 6 hours to be detected [33]. With Dynasore pretreatment, the early cardiac efflux of cTnI was decreased 70% (at 30 minutes of reperfusion), further confirming the protective effects of Dynasore on cardiac muscle.

### In Cultured Cardiomyocytes, Dynasore Increases Cell Survival and Viability

**Figure 4:**
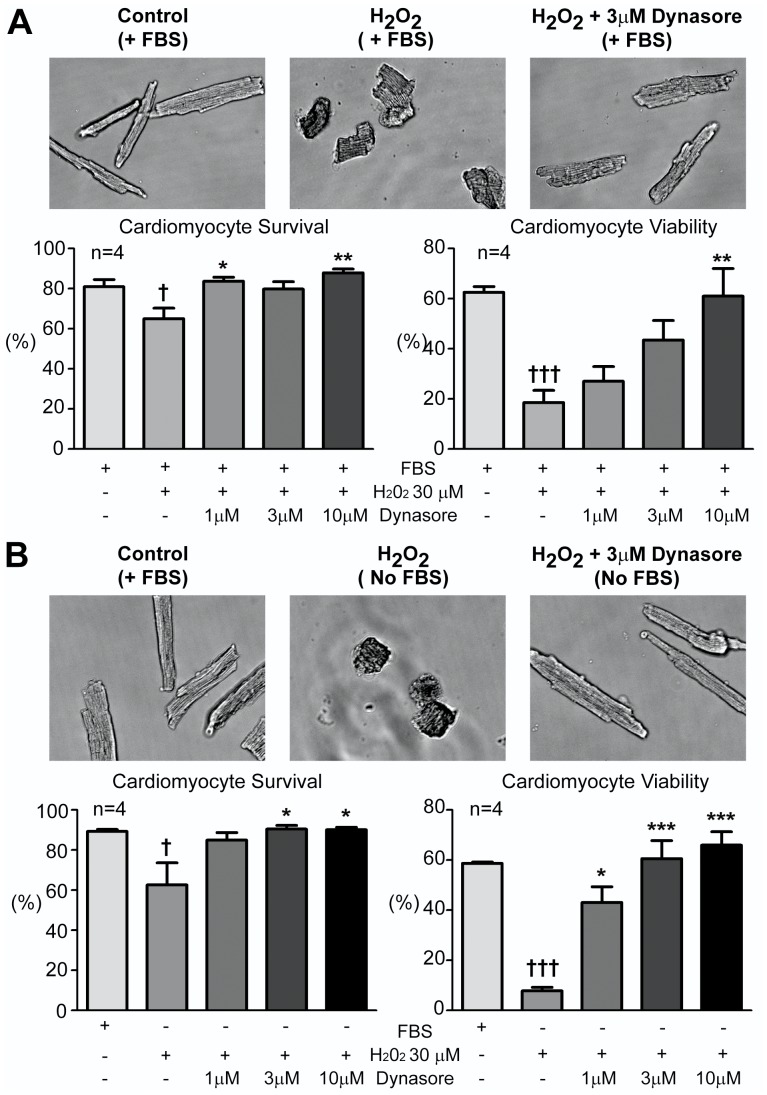
Dynasore increasses cardiomyocyte survival and viability. Trypan blue exclusion assay was used to identify cell survival and viability of cardiomyocytes subjected to oxidative stress in the presence (A) and absence of FBS (B). A. Dynasore increases cell survival and viability in oxidative stressed (exposed to 30µM H_2_O_2_ for 35 min) cardiomyocytes. Top, representative phase images of cardiomyocytes. Bottom, summarized cell survival and viability results. B. Dynasore further increased cell survival and viability in serum depleted and oxidative cardiomyocytes. Top, representative phase images of cardiomyocytes. Bottom, summarized cell survival and viability results. (** *P*<0.01, ***P<0.001 when compared to stressed cardiomyocytes without Dynasore treatment; † *P*<0.05, ††† *P*<0.001 when compared to non-stressed cardiomyocyte controls).

The data in Figures 2 and 3 involved protocols of complete no-flow ischemia with whole heart preparations. We were interested if the protective effects of Dynasore extend to a more traditional cellular cardiomyocyte assay with a more controlled stress insult. Isolated adult mouse cardiomyocytes in culture were subjected to oxidative stress by addition of hydrogen peroxide in the presence or absence of serum. Healthy adult cardiomyocytes have a typical elongated rod shape. However oxidative stress significantly damage cell morphology resulting in a well described contracted morphology [27]. We observed significant cardiomyocyte contraction with oxidative stress to 30 µM H_2_O_2_ (Figure 4). Note that Dynasore prevents cardiomyocyte morphological changes induced by oxidative stress.

Cell survival and viability can further be assayed by TBE and cell morphological changes, respectively (see Methods). As seen in the bar graphs of Figure 4, as expected, oxidative stress causes a significant reduction in both cell survival and viability. However the presence of Dynasore in the culture medium results in significantly improved survival and a major improvement in viability. The beneficial effects of Dynasore on viability and survival were dose dependent and are also more prominent in serum free conditions (Figure 4B).

### Dynasore Preserves Cellular ATP Content in Stressed Cardiomyocytes

**Figure 5:**
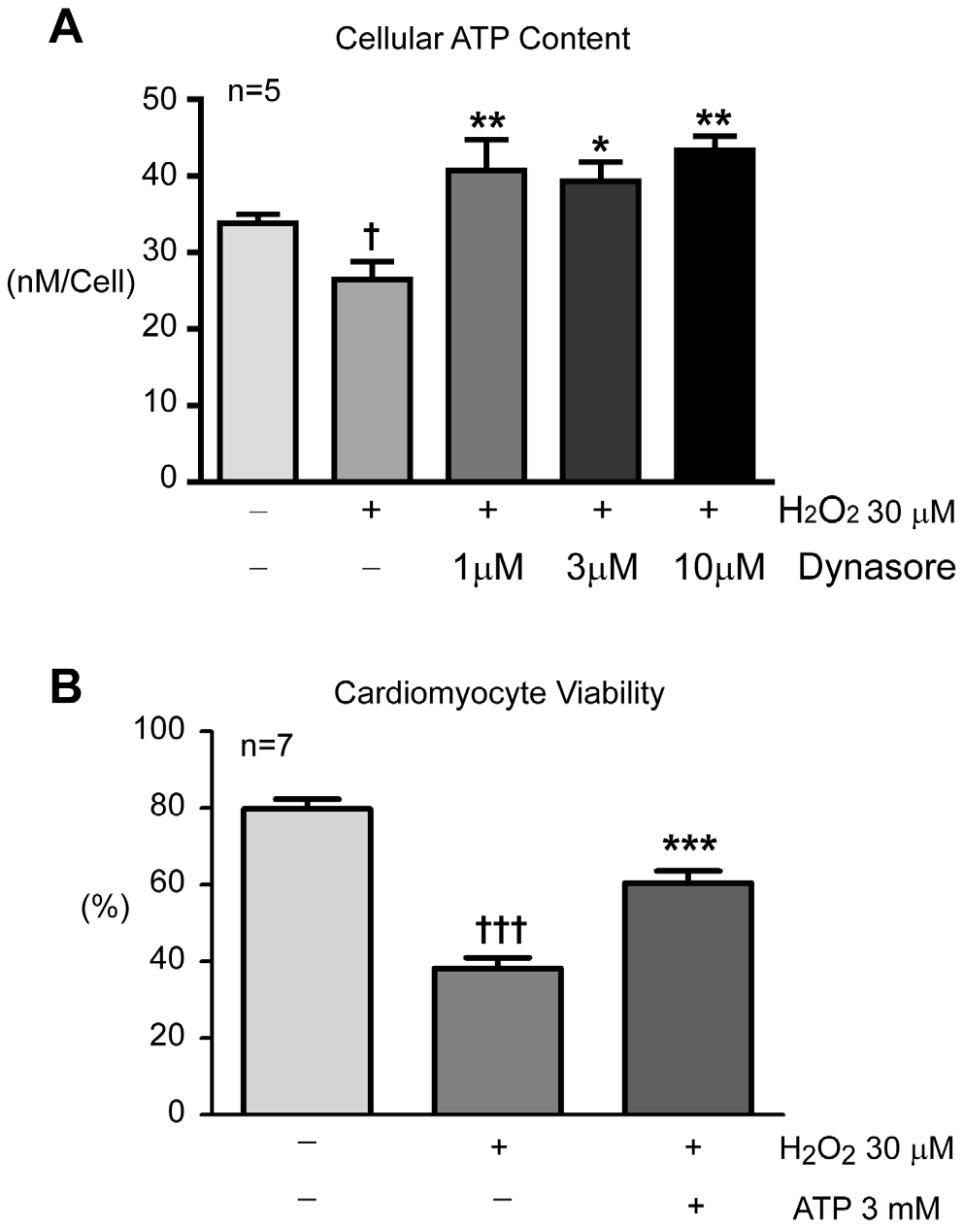
Dynasore preserves cellular ATP content in stressed cardiomyocytes. A. Dynasore preserves cardiomyocyte ATP content. Adult mouse cardiomyocytes were exposed to 30µM H_2_O_2_ for 35 min in the absence and presence of Dynasore. Cellular ATP content (total ATP normalized to amount of surviving cardiomyocytes) were calculated and compared among the different treatment groups. B. Direct supplement of ATP (3 mM in culture medium) increases cardiomyocyte survival after H_2_O_2_ exposure. (**P<0.01, ***P<0.001 when compared to H_2_O_2_ stressed cardiomyocytes without Dynasore treatment; †*P*<0.05, †††*P*<0.001 when compared to control cardiomyocytes).

Dynasore’s beneficial effect on cell fate and the ameliorating effect of serum (Figure 4) suggest that the mechanism of Dynasore’s effect is related to energetics. This hypothesis was tested by recording cellular ATP content in oxidative stressed cardiomyocytes. To control for different amount of living cardiomyocytes per culture well, net ATP content was normalized to the amount of surviving cardiomyocytes counted per dish. As seen in Figure 5A, as low as 1 µM dose of Dynasore increases cellular ATP content in live cardiomyocytes. To confirm that Dynasore induced higher levels of ATP are protective, we performed an ATP rescue experiment and found, in Figure 5B, that direct supplementation with exogenous ATP has similar effect to Dyansore on cardiomyocyte viability subjected to oxidative stress.

**Figure 6:**
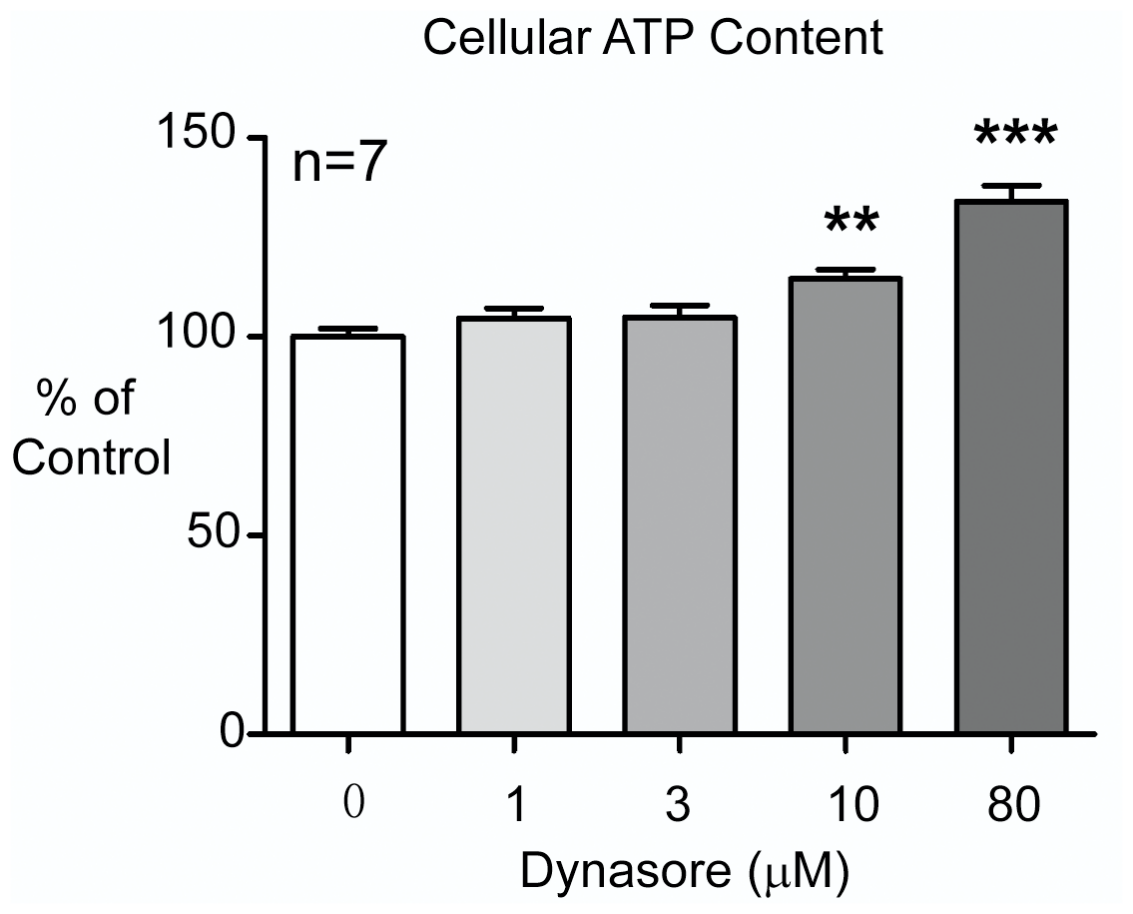
Effects of Dynasore on cellular ATP content in unstressed Hela cells. Low dose Dynasore does not change cellular ATP content in unstressed Hela cells, whereas high dose of Dynasore (>10 µM) increases ATP content. Cellular ATP content were calculated and compared among the different treatment groups. (*P<0.05, **P<0.01 when compared to control cells without Dynasore treatment).

Next, we explored whether Dynasore simply preserves ATP during stress or can actually generate ATP independent of a metabolic insult. Since the process of dissociating and culturing adult mouse cardiomyocytes is already a stress to these primary cells, a typical cell line of Hela cells in standard cell culture hemostasis was used in this analysis. As seen in Figure 6, low dose Dynasore (1–3 µM) has no effect on cellular ATP content, indicating that Dynasore may preserve ATP in stressed cells rather than be responsible for ATP production. Note high dose Dynasore (>10µM) increases cellular ATP content, possibly indicating direct ATP production or, more likely, the energetic benefit of limiting Dynamin GTPase dependent endocytosis at higher dosage.

### Dynasore Inhibits Oxidative Stress-induced Mitochondrial Fission

**Figure 7:**
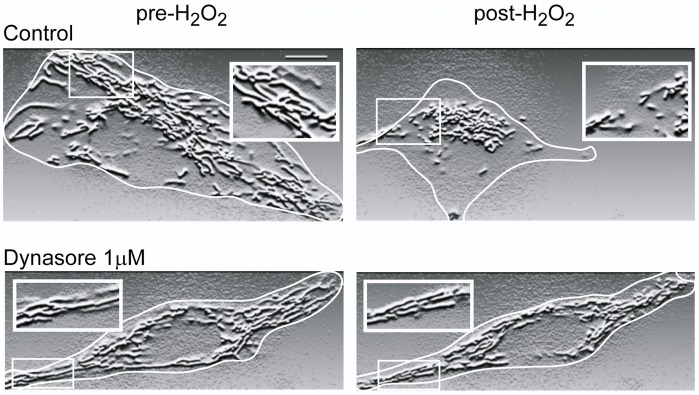
Dynasore prevents oxidative stress-induced mitochondrial fission. Cultured human Hela cells were used for mitochondrial morphology study. Top, Hela cells have elongated connected mitochondrial network (left), which was fragmented after oxidative stress (right). Bottom, 1 µM Dynasore pretreatment prevents oxidative stress-induced mitochondrial fragmentation.

Given the reported inhibitory effects of Dynasore on Drp1 [19] and the observed effect on increasing cell survival and viability (Figure 4) as well as preserving cellular ATP content (Figure 5), we hypothesized that the cardioprotective effect of Dynasore (Figure 2 and 3) is mediated by inhibition of Drp1 dependent mitochondrial fission. To test this hypothesis, cultured cells were transduced with baculovirus expressing a mitochondrial targeted fluorescent protein. Instead of adult mouse cardiomyocytes, HeLa cells were used for this study because the relatively flat HeLa cell morphology is permissive to detailed high resolution mitochondrial imaging. The cardiac atrial origin HL-1 cells and neonatal cardiomyocytes are not used for this study due to their resistance to oxidative stress [34], probably related to differential mitochondrial fusion-fission dynamics. The morphology of mitochondria was examined by spinning disc confocal microscopy before and after the exposure to 200 µM H_2_O_2_ for 15 minutes. As seen in Figure 7, the non-stressed mitochondria have an elongated and well-organized reticulum network, whereas oxidative stress induces mitochondrial fragmentation. Note the cellular morphology is also altered after oxidative stress, resulting in contracted and smaller cells. Consistent with the above hypothesis, pretreatment of the cells with Dynasore prevented oxidative stress induced mitochondrial fission (Figure 7, bottom row), retaining the original organized mitochondrial reticulum network and maintaining normal cell morphology.

## Discussion

We have found that the small dynamin-GTPase inhibitor Dynasore protects mitochondria and significantly benefits cardiac lusitropy in hearts subjected to I/R injury. The cardioprotective effect is observed in both *ex vivo* perfused mouse heart preparations and isolated cultured cardiomyocytes. Dynasore also, in dose dependent fashion, increases cell survival in cultured primary adult mouse cardiomyocytes exposed to oxidative stress. In the surviving cells, Dynasore preserves cellular ATP content whereas adding exogenous ATP provides a similar rescue in the absence of Dynasore. Of note, the cardioprotective dose of Dynasore is significantly lower than the dose used to effectively block endocytosis.

### A Novel Cardiac Lusitropic Role of Dynasore

The small molecule Dynasore was identified as an endocytosis inhibitor seven years ago [19]. It was found to be a non-competitive inhibitor of the GTPase activity of dynamin1, dynamin2, and the mitochondrial pro-fission dynamin isotype Drp1 (Cartoon in Figure 1). By blocking plasma membrane dynamin1 and dynamin2, Dynasore acts as a potent blocker of dynamin-dependent coated vesicle formation, resulting in stabilization of intermediates including U-shaped and O-shaped pits [19]. Since its discovery, Dynasore has been used as an effective cardiac related endocytosis inhibitor due to its potency and limited cytotoxicity [34], [35].

Since Dynasore inhibits Drp1 *in vitro* [19], it is interesting that Dynasore significantly prevents LVEDP elevation during I/R injury without affecting LVDP (Figure 2) indicating a novel lusitropic effect. Diastolic dysfunction is usually associated with prominently altered nucleotide levels [36] or ATP turnover and catabolism [37]. Therefore the beneficial lusitropic effect may be mediated by Dynasore’s ability to preserve the ATP reserve in stressed cardiomyocytes (Figures 5). Dynasore inhibits GTPase activity at both the plasma membrane (Dynamin 1, 2) and the mitochondria membrane (Drp1) [19]. However, the current lusitropic dose of Dynasore (1 µM, Figure 2) is significantly lower than the previously reported inhibitory dose of Dynasore (IC50 ∼15 µM) on dynamin dependent endocytosis [19], indicating an effect separate from dynamin inhibition at the plasma membrane. The low non-endocytosis related dose of Dynasore that blocks oxidative stress-induced mitochondrial fission (Figure 7) is most likely due to Drp1 inhibition [18], [38].

Interestingly, Dynasore improves diastolic function with acute I/R injury, without affecting systolic function (Figure 2). It might be that no-flow ischemia permits the accumulation of toxic metabolites, such as low pH, which can limit inotropy independent of myocardial energetics. However inotropy was unaffected by Dynasore during the reperfusion period which should wash out toxic metabolites, even while lusitropy was preserved (Figure 2). Therefore lusitropy specificity could result from diastolic function being a more sensitive indicator of cardiac ATP and other mitochondrial dependent energy production.. In healthy individuals, mild hypoxia results in diastolic dysfunction without affecting systolic function [39]. Diastolic sensitivity to hypoxia could be due to a decline in high energy phosphate metabolism [40]. Advanced age is also a well established association with progressive diastolic dysfunction. Hypoxia and age both result in irreversible damage to mitochondrial genes involved in oxidative phosphorylation [41]. These mutations limit mitochondrial function and could contribute to progressive diastolic dysfunction [42]. It is possible that rescue of oxidative phosphorylation could be beneficial to not just acute diastolic dysfunction, but chronic diastolic dysfunction as well.

### Mitochondrial Morphology and Cardiomyocyte Survival

In non-stressed conditions, mitochondria fusion prevails resulting in elongated, tubular, and interconnected mitochondria networks. During ischemia, the dynamic balance shifts from fusion to fission, resulting in fragmented and discontinuous mitochondria [18], [43], as well as mitochondrial outer membrane permeablization, release of apoptotic factors, and activation of apoptosis. Apoptotic cell death is understood to be a major contributing factor of I/R injury [44]. Recent studies show that alteration of mitochondrial morphology is significant in ischemic hearts [18], [43] and modulation of which can protect the heart against I/R injury [18].

The apoptotic cell death that results from mitochondrial fission and fragmentation is mediated by activation of a key mitochondrial pro-fission protein Drp1 [38], [45]. Recently, it was reported that a dominant-negative mutant of Drp1 induces mitochondrial elongation and pharmacological inhibition of Drp1 protects against I/R injury in the heart [18]. We found in human cells that Dynasore significantly prevents stress induced mitochondrial fragmentation and maintains a normal elongated mitochondrial morphology (Figure 7). Drp1 is a known target of Dynasore, and it follows that the mitochondrial protection of Dynasore is mediated by inhibition of Drp1. Future studies could explore the effect of Dynasore on activities of other mitochondrial fusion and fission related regulators, such as MFN1, MFN2, OPA1, FIS1, MFF.

In conclusion, our study provides the first evidence that Dynasore has a potent lusitropic effect during I/R injury. The mechanism is mitochondrial protection and preservation of oxidative phosphorylation. Pharmaceutical therapy that preserves mitochondrial function may not just benefit myocardial survival, but improve diastolic dysfunction as well.
